# Convolutional Neural Network-Based Approach for Cobb Angle Measurement Using Mask R-CNN

**DOI:** 10.3390/diagnostics15091066

**Published:** 2025-04-23

**Authors:** Marcos Villar García, José-Benito Bouza-Rodríguez, Alberto Comesaña-Campos

**Affiliations:** 1 Department of Design in Engineering, University of Vigo, 36208 Vigo, Spain; jbouza@uvigo.gal; 2 Design, Expert Systems and Artificial Intelligent Solutions Group (DESAINS), Galicia Sur Health Research Institute (IIS Galicia Sur), SERGAS-UVIGO, 36213 Vigo, Spain

**Keywords:** scoliosis, Cobb angle measurement, Mask R-CNN, vertebrae detection and segmentation

## Abstract

**Background:** Scoliosis is a disorder characterized by an abnormal spinal curvature, which can lead to negative effects on patients, affecting their quality of life. Given its progressive nature, the classification of the scoliosis severity requires an accurate diagnosis and effective monitoring. The Cobb angle measurement method has been widely considered as the gold standard for a scoliosis assessment. Commonly, an expert assesses scoliosis severity manually by identifying the most tilted vertebrae of the spine. However, this method requires time, effort, and presents limitations in measurement accuracy, such as the intra- and inter-observer variability. Artificial intelligence provides more objective tools that are less sensitive to manual intervention aiming to transform the diagnosis of scoliosis. **Objectives:** The objective of this study was to address three key research questions regarding automated Cobb angle quantification: “Where is the spine in this radiograph?”, “What is its exact shape?”, and “Is the proposed method accurate?”. We propose the use of Mask R-CNN architecture for spine detection and segmentation in response to the first two questions, and a set of algorithms to tackle the third. **Methods:** The network’s detection and segmentation performance was evaluated through various metrics. An automated workflow for Cobb angle quantification and severity classification was developed. Finally, statistical methods provided the agreement between manual and automated measurements. **Results:** A high segmentation accuracy was achieved, highlighting the following: mIoU of 0.8012, and a mean precision of 0.9145. MAE was 2.96° ± 2.60° demonstrating a high agreement. **Conclusions:** The results obtained in this study demonstrate the potential of the proposed automated approach in clinical scenarios, which provides experts with a clear visualization of each stage in the scoliosis assessment by overlaying the results onto the X-ray image.

## 1. Introduction

**Figure 1:**
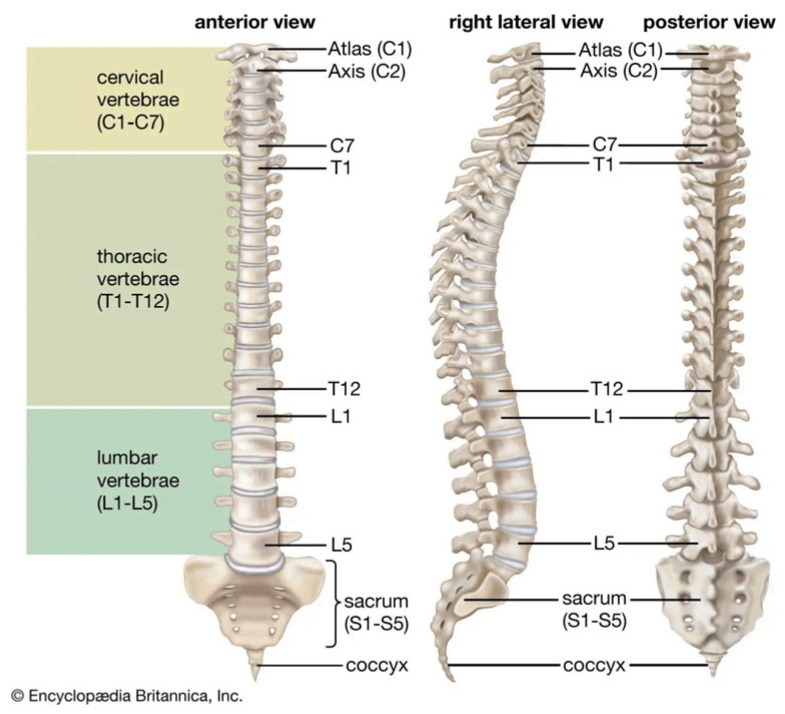
The vertebral column [6].

Scoliosis is a condition of lateral deviation of the spine with a curvature greater than 10 degrees [1,2]. Additionally, the vertebrae may twist around their axis, presenting torsion that add complexity. As shown in Figure 1, the human vertebral column is divided into five regions, which are structured from bottom to top as follows: coccyx, sacrum, lumbar vertebrae, thoracic vertebrae, and cervical vertebrae. Of the total number of vertebrae, 5 of them (L1–L5) belong to lumbar region, and 12 (T1–T12) are located in the thoracic region. Scoliosis assessment is performed by measuring the spinal curvature in degrees. Different authors use the following classification to evaluate the severity of the scoliosis: mild scoliosis is considered at 10–20 degrees; moderate between 20 and 40 degrees; and severe above 40 degrees [2,3]. Other studies have used different threshold values to define curvature degrees. In the treatment of scoliosis there are several levels of severity and ways to deal with it. An early and precise diagnosis allows selection and planning the most suitable treatment. In an initial evaluation, doctors rely on clinical methods with no patient radiation exposure. Observational tests to detect suspect scoliosis include assessment of trunk rotation, the Adams forward bend test, and spine measurement instruments [4]. Through a physical examination, the specialist looks for asymmetries in the patient’s back. When clinical examination suggests an abnormal curvature of the spine, anteroposterior X-ray images, considered the gold standard, are used for scoliosis assessment [5].

Radiography experts measure the Cobb angle in the X-ray image by using instruments such as a goniometer. They find the most tilted vertebrae and quantify the angle of the spinal curvature directly onto the radiograph. This manual method requires time and effort. Manual measurements present a large intra- and inter-observer variability due to the wide range of the scoliotic curvature among patients, measurement errors, and low image quality [7]. Moreover, the correct definition of anatomical landmarks, such as end vertebrae and tilted vertebrae, is essential to guarantee precise measurements and to reduce subjective errors associated with their manual identification [8,9]. Manual measurements depend on expert experience and judgment. These limitations can negatively impact the accuracy of measurements, opening opportunities for the development of methods that ensure the correct diagnosis. This research introduces an automated approach based on a convolutional neural network to reduce the intra- and inter-observer variability in the scoliosis severity assessment through Cobb angle measurement. To this end, we propose the use of Mask R-CNN [10] for object instance segmentation in X-ray images. This method predicts a binary mask for each candidate object. Due to this particular capability, we employ Mask R-CNN for single-instance segmentation of the spine, generating a mask that replicates the scoliotic curvature. This mask is then processed using an algorithm that extracts the midline of the scoliotic curvature by connecting the midpoints derived from the spine’s boundary. The algorithm further identifies anatomical landmarks, calculates the Cobb angle, and classifies the severity of the spinal curvature. Additionally, it provides a clear visualization of each stage in the measurement process, which can improve the interpretability of the results.

The article is divided into six sections. In Section 2, a review of related works is provided. Section 3 describes the materials and methods used in the study. Section 4 details the experimental analysis and results. Section 5 discusses the study and its scope. Finally, Section 6 presents the conclusions of the study and future work.

## 2. Related Works

Precision in spinal curvature assessment is required to guarantee precise diagnosis. Several parameters directly impact accuracy and repeatability of measurements such as manual intervention, observer expertise, image quality, and assessment criteria. Variability in inter- and intra-observer assessment led to the development of more objective tools, less sensitive to manual intervention, to improve reliability scoliosis evaluation. The Cobb angle method, which quantifies the degree of spinal curvature, has been considered the reference standard for its assessment [3,11,12,13,14,15]. Wiliński et al. [5] demonstrated that the Cobb angle was the most reliable method through a comparison of measurements performed by inexperienced users employing the Cobb, Centroid, and Ferguson techniques.

In 2009, Vrtovec et al. [16] highlighted the advantages of using image modern processing techniques, which help experts in improving diagnostic and therapeutic planning for spinal conditions. Jin et al. [17] addressed challenges such as the identification accuracy and computational time in computer vision and deep learning. The use of techniques to improve the quality of medical images has increased in recent years, aiming to facilitate the Cobb angle measurement. Despite the good results, there are several limitations in the field. Landmark and segmentation approaches are the most used for Cobb angle quantification [14]. In this study, we have reviewed both vertebral landmark prediction [3,4,8,9,11,12,13,15,18,19,20,21,22,23,24,25,26,27,28] and vertebrae segmentation [2,14,29,30,31,32,33,34,35,36,37,38]. However, those approaches that identify vertebrae individually are not sufficiently accurate [39]. According to Gstoettner et al. in 2007 [8], the selection of the superior and the inferior end vertebrae is a difficult task and represents the main sources of error. The effect of applying filters to improve endplate identification in X-ray images with low-resolution was studied by Anitha et al. [9] in 2014. In 2017, the relationship between anatomical landmarks and the Cobb angle was addressed using structured support vector regression (SSVR) by Sun et al. [18]. Wu et al. [19] proposed a deep learning approach that combined the CNNs’ capabilities for feature extraction and statistical methods. These authors also studied the effect of occlusion in Cobb angle estimation using anteroposterior and lateral views of the spine. In 2019, Chen et al. [21] used two networks separately to identify anatomical landmarks and for Cobb angle measurement. Yang et al. [22] identified vertebrae from the thoracic and lumbar region as critical using two convolutional architectures, Faster-RCNN and ResNet 101. An order that facilitates learning, first by detecting vertebrae, followed by locating landmarks, was proposed by Khanal et al. [11]. Cerqueiro et al. [24] explored automated estimation of the Cobb angle using image processing and active contour algorithms to identify the spine contour. However, the snake model can lose features in regions with poorly defined boundaries, particularly when vertebral bodies overlap each other, as noted by Jin et al. [17] and Hoblidar and Prabhu [29]. Given the high performance of both segmentation and landmark estimation, several authors addressed Cobb angle quantification by combining these deep learning techniques. However, these deep learning techniques are commonly used in combination. In 2021, Fu et al. [12] proposed an architecture aiming to combine segmentation with landmark estimation. A study aiming to evaluate variations in the measurements performed by different observers through the application of Cobb, Ferguson, Diab, and Centroid methods was conducted by Thalengala et al. [3], who emphasized the importance of evaluating the inclination of vertebral bodies. In 2022, Huang et al. [13] proposed the use of artificial neural networks and oscillograms that represent endplate inclinations to calculate the Cobb angle. Sun et al. [25] addressed the Cobb angle measurement in X-ray images through two sequential operations: vertebrae segmentation and the application of crop and zoom to locate the corners of each vertebra. In 2023, Han et al. [4] proposed a machine learning algorithm that quantifies scoliosis by detecting maximum pixel values, and fitting a spline curve through anchor points to draw the spinal curvature. The CNN method proposed by Maeda et al. [26] consisted of thoracic and lumbar region identification, and the detection of the four corners of each vertebra as anatomical landmarks. The points identified as the most tilted vertebrae were used to measure the Cobb angle. Qiu et al. [27] addressed noise complex computational operations by introducing a novel deep learning approach which generates spine region, centerline, and the boundary as multiple sources of morphological information. Suri et al. [28] reported a neural network architecture that enables Cobb angle measurements even when the presence of hardware obstructs parts of the spine. In 2024, Chui et al. [15] employed a feedforward neural network (FNN) architecture for Cobb angle prediction and spinal curvature progression analysis. Using a landmark detection algorithm, they identified the center and the edge points of each vertebra to calculate the angles. Maharasi et al. [40] proposed the use of the U-Net architecture to evaluate the scoliosis stage through the detection of several anatomical landmarks. Most automated methods use the superior and inferior endplate regions of each vertebra, commonly named as vertebral plates, to calculate the Cobb angle based on the most tilted vertebrae, known as the end vertebrae. However, the identification of these end vertebral plates remains the main source of error. To address this challenge, Hoblidar and Prabhu [29] proposed an automatic vertebrae segmentation system using image processing techniques to ensure the identification of the most tilted vertebrae at the superior and inferior ends of the spinal curvature. In 2019, Horng et al. [2] applied the original U-Net architecture with two of its variants, Residual U-Net and Dense U-Net, to improve vertebrae segmentation performance on anteroposterior (AP) spinal X-ray images. Nevertheless, selecting an appropriate novel and training configuration still requires human knowledge, as noted by Vuola et al. [31]. These authors compared two widely used segmentation approaches, U-Net and Mask R-CNN, in a nuclei segmentation task to evaluate their strengths and limitations. While Mask R-CNN is better at detecting objects by predicting bounding boxes, U-Net is more precise at segmentation tasks. Pan et al. [41] employed two Mask R-CNN approaches: one trained for spine segmentation and the other designed for vertebrae segmentation. Convolutional neural networks are the most used architectures for object detection and classification in images, according to Alharbi et al. [32]. In 2020, these authors used a pretrained ResNet50, to detect vertebrae in spine X-rays. In 2021, Zhang et al. [33] included sacral vertebrae in their study, providing an efficient and accurate solution for whole-spine vertebra segmentation. In 2022, Caesarendra et al. [14] introduced the concept of intervertebral displacements for Cobb angle quantification using a convolutional neural network. Zhao et al. [34] improved the U-Net architecture by generating a binary segmentation map of each vertebra and identifying the most tilted candidates to calculate the Cobb angle. In 2023, Wong et al. [35] developed an AI-based algorithm that combined two convolutional neural networks: the first for spinal column segmentation, and the second for vertebral bodies’ segmentation and bounding box extraction. The tilted angles of these boxes were used to compute the Cobb angle. In 2024, Low et al. [36] developed a deep learning model using a two-stage approach; an attention-based deep neuronal network to segment and identify individual vertebrae, followed by polynomial curve fitting to the vertebral centroids for Cobb angle calculation. Rahmaniar et al. [38] proposed a convolutional neural network-based approach that integrates vertebrae segmentation and hourglass modules to enhance landmark localization by detecting five coordinates per vertebra (center and four corners), which are used to identify the most tilted vertebrae.

In this section, different studies related to this article have been reviewed. Image feature extraction and segmentation capabilities of convolutional neural networks have been demonstrated. Image processing techniques and combinations of networks are widely employed in the state of the art, aiming to calculate the Cobb angle and severity scoliosis. Key features are detected using novel strategies based on the location of the anatomical landmarks and vertebral segmentation with the objective of detecting the most tilted vertebrae, which remains a challenge and is the basis for Cobb angle quantification. Precision in spine detection or vertebrae segmentation is essential in scoliosis assessment. Mask R-CNN incorporates the prediction of a binary mask for each region of interest (RoI); in our case, a single binary mask is generated for the spine. This particular characteristic is relevant in our decision. Our motivation is focused on the use of Mask R-CNN as a single-instance segmentation approach to predict the mask of the spine and use the spinal boundary as the basis for our scoliosis assessment approach. However, the reviewed studies do not utilize the additional information provided by the Mask R-CNN network in the form of a mask to draw the midline of the spine and, through the analysis of its curvature, identify key anatomical landmarks that enable a simplified calculation of the Cobb angle based on the most tilted vertebrae and the assessment of the scoliosis severity. We offer to the users the visualization and recording of the workflow, displaying the main results of each step, as support for improving the interpretability of the scoliosis assessment procedure, to understand the prediction and collect data for further comparison. In the following section, we describe the experimental setup of this study.

## 3. Materials and Methods

In this study, the performance of the Mask R-CNN method [10] was analyzed for object detection and segmentation. Our approach was designed with the aim of identifying the spine as a single instance within anteroposterior X-ray images. To this end, we used a network that generates the mask corresponding to the spine. Various metrics were employed to validate the network’s reliability and robustness in spine segmentation. Considering the segmentation accuracy, different algorithms were developed to identify anatomical landmarks, enabling the Cobb angle quantification and severity classification. Additionally, we developed a visual interface where users can follow the main steps of the process and view the results at each stage by uploading the X-ray images.

### 3.1. Materials

The collection of spinal images was obtained from the public AASCE MICCAI 2019 dataset [42], which contains 98 raw anteroposterior (AP) spine X-ray images with different dimensions, ensuring a standardized source of data. Mask R-CNN was implemented for object detection and segmentation in a virtual environment using Python 3.12.7, TensorFlow 2.16.2, and Keras 3.7.0. The model was trained locally on a GeForce RTX 4080 Laptop GPU (12 GB) with an Intel i9-14900HX processor (Intel Corporation, Santa Clara, California, USA). Statistical analyses were conducted using the Python libraries NumPy, Pandas, and Pingouin. The algorithms were developed in the JupyterLab environment, generating multiple outputs, including Cobb angle calculation, scoliosis severity classification, spine landmarks, and clear representations of the results on the input X-ray image.

### 3.2. Methods

The method proposed in this study provides a systematic approach that enables the evaluation of spinal curvature severity based on vertebral alignment. In response to the questions “Where is the spine in this radiograph?” and “What is its exact shape?”, Mask R-CNN addresses both: the bounding box localizes the spine within the image (where), and the segmentation mask defines its exact shape (what). The third question, “Is the proposed method accurate?”, is answered by evaluating the error between the measurements performed by the two observers (ground truth) and those obtained using our approach.

**Figure 2:**
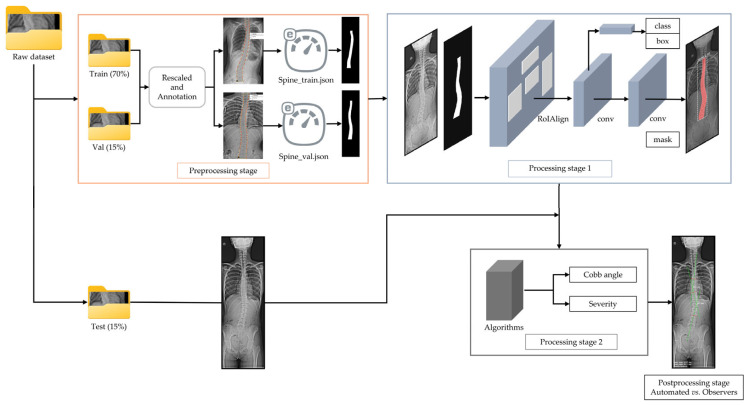
Overview of proposed workflow. First, the raw images (training and validation subsets) are rescaled to a height of 2000 pixels, and the spine contour (the label) is annotated during the preprocessing stage as part of dataset construction process. In the processing stage 1, the network learns to identify the spine based on the annotated binary mask. Processing stage 2 involves Cobb angle quantification and scoliosis severity assessment. At this stage, the input from the test subset is automatically rescaled to a height of 2000 pixels. Finally, in the postprocessing stage, automated measurements are compared with those provided by the human observers.

To answer these questions, we developed a four-stage workflow based on the raw dataset: (1) a preprocessing stage, including image rescaling and annotation; (2) network processing stage; (3) Cobb angle and severity classification processing stage; and (4) postprocessing stage for error evaluation. The workflow design is illustrated in Figure 2.

In the following subsections, a detailed explanation of each stage comprising the proposed workflow is provided.

#### 3.2.1. Preprocessing Stage

Before the preprocessing stage, 70% of the raw images were assigned to the training set, with the remaining 30% equally divided between validation and testing sets. This proportion was selected to ensure a sufficient number of images for model learning. The images in each subset were distributed randomly to minimize selection bias.

The network was trained using only the spine class label plus background. To this end, X-ray images from both the training and validation subsets were employed. Accordingly, the output of the model was limited to the spine localization and mask generation, while the degree of curvature was calculated during the inference stage using a set of algorithms, thereby enabling Cobb angle quantification and scoliosis severity assessment. The original spine X-ray images corresponding to training and validation subsets were rescaled to a height of 2000 pixels. Manual annotation of these images was performed using VGG Image Annotator (VIA) software, version 2.0.12. This software allows exporting annotations to a JSON file with the coordinates of the spine boundary. During this stage, we annotated the contour of the spine using points aiming to detect the spine and mask generation. The boundary of the spine comprised the ground truth (GT) of our dataset and was used to train the model. The objective of this labelling phase was to prepare the training and validation dataset. The spinal contour was identified and annotated in each image. We used VIA to place points along the boundary of the spine in each radiograph. The coordinates of these points that define the contour of the spine in the training and validation dataset were stored in a JSON file. These annotations were used to replace each X-ray by its binary mask. The pixels of the spine were marked in white, and the rest are marked in black. This ensured the availability of the training labels required by the Mask R-CNN and also enabled the model to distinguish the region corresponding to the spine. This stage allowed the network to learn the shape of the spine and its spatial arrangement during the training process. Mask R-CNN requires a binary mask as the training label associated with the X-ray image from which it was generated.

We used skimage.draw.polygon function to convert these coordinates into a binary mask suitable for the network. Although the original radiographs were greyscale (single-channel), the Mask R-CNN architecture expects input in RGB format (three channels); therefore, a conversion from greyscale to RGB was performed. In this operation, the anatomical information is preserved while an image visual identical and compatible with the model is created. The JSON file, which stores the manually annotated coordinates of the spinal contour, is used to generate the binary masks that serve as training labels. These labels were generated once for the entire dataset. A dataset is specifically created for identify the region of the spine, without considering the severity of the idiopathic condition, or even whether it is present. The network does not aim to discriminate or classify possible cases of scoliosis, only to identify the spinal region. For this reason, this process is mandatory, as it constitutes the training and validation dataset for the network. However, once this dataset has been annotated, the process does not need to be repeated to analyze new radiographs for testing or future data. In such cases, only the input radiographs must be rescaled.

While the raw images were specifically used to train the model for spine detection and segmentation, we also analyzed the degree of spinal curvature to extract additional information from the dataset that may be relevant for future work and necessary for error evaluation on the test subset. We used the severity classification provided by Horng et al. [2]. The raw dataset was distributed as follows: 52% of the cases were classified as moderate, 28% as mild, 13% as severe, and 7% as spinal curve (i.e., absence of scoliosis). The authors noted the dataset presents an imbalanced distribution based on the values of the Cobb angle. Although this imbalance may introduce a morphological bias, favoring more frequently curvatures, this study does not constitute a multiclass annotation problem.

#### 3.2.2. Processing Stage 1: Network Training

In this stage, the convolutional neural network is training for spine detection and segmentation task, using the labels annotated during the previous stage. As mentioned, Mask R-CNN requires a binary mask for training. We used the skimage.draw.polygon function to convert the coordinates of the spine boundary stored in the JSON file into a binary mask suitable for the network. Although the original radiographs were greyscale (single-channel), the Mask R-CNN architecture expects input in RGB format (three channels); therefore, a conversion from greyscale to RGB was performed.

A brief explanation of the model pipeline is provided as follows: The Region Proposal Network (RPN), a core component of the network, generates candidate regions of interest (RoIs) with a high probability of containing the spine. Then, it predicts anchors with a probability score indicating the presence of the spine (binary classification: spine/background). Finally, the top proposal’s RoIs are selected and passed through fine classification, bounding box regression, and mask segmentation.

The training subset was used to fit the model, while the validation subset was employed in the optimization process. The test subset was reserved for the next stage. We optimized hyperparameters through multiple trials using Optuna, with training monitored by callbacks such as EarlyStopping and CSVLogger. The objective of this stage was to minimize validation loss and identify the best epoch for spine detection and segmentation.

**Figure 3:**
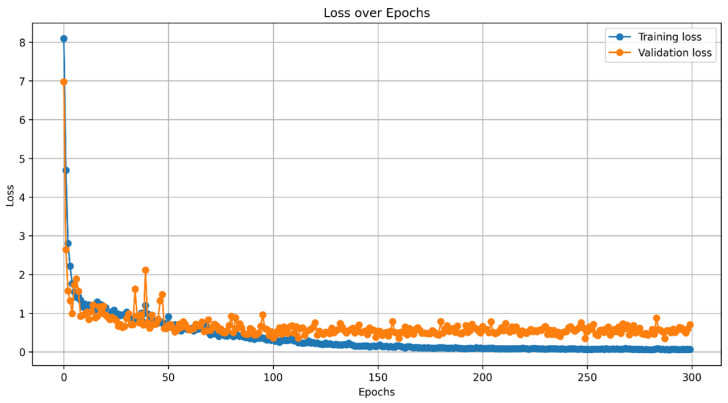
Training and validation loss function over epochs.

To this end, we evaluated the performance of the Mask R-CNN network through a comprehensive analysis of the training process. We implemented the Mask R-CNN architecture using the GitHub repositories referenced in [43,44], specifically employing the implementation based on TensorFlow 2.0. As mentioned in the Materials subsection, the dataset comprised 98 anteroposterior spine X-ray images, split into 70% for training, 15% for validation, and 15% for testing. We employed transfer learning by applying COCO pre-trained weights and fine-tuning the model for spine segmentation in X-ray images. Preliminary experiments guided a hyperparameter optimization phase, where various fine-tuning strategies were explored. Multiple training sessions were conducted, monitoring training and validation loss curves to refine model performance. No data augmentation techniques were applied during training. The final model was trained for 300 epochs, demonstrating consistent performance throughout the training process, as illustrated in Figure 3.

**Figure 4:**
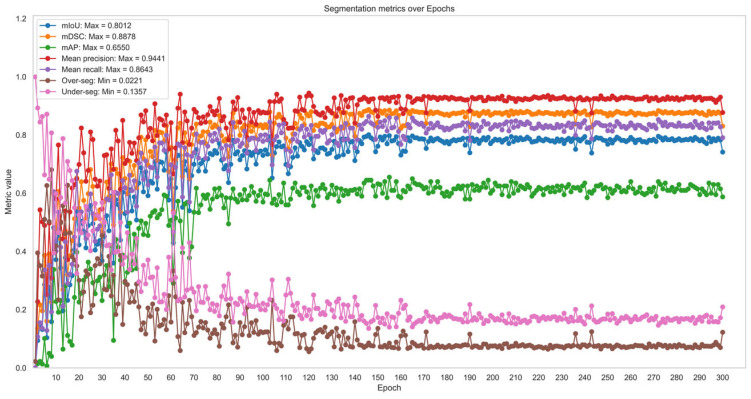
Evolution of segmentation metrics over 300 epochs.

As shown in the previous figure, readers may observe that the validation loss function seems to reach a stable performance trend after approximately 50 epochs. In this study, the authors decided to extend and monitor the training process to achieve the best segmentation results and define the most appropriate epoch. Figure 4 presents the evolution of the segmentation metrics, which supports the decision to extend the monitoring beyond 50 epochs, up to 300 epochs.

The segmentation model was evaluated using complementary metrics: intersection over union (IoU), also referred to as the Jaccard index, evaluates the overlap between the predicted mask and the ground truth mask; dice similarity coefficient (DSC) reflects the global similarity between the segmented regions and the reference; average precision (AP) serves as an overall performance indicator in terms of detection and precision of segmented regions; precision indicates how many of the positives predicted by the model were actually correct; and recall quantifies how many true positives were successfully detected by the model. Finally, over-segmentation occurs when the model predicts a region larger than the actual object, including areas that do not belong to the target, and under-segmentation happens when the predicted region is smaller, missing relevant parts of the target.

**Figure 5:**
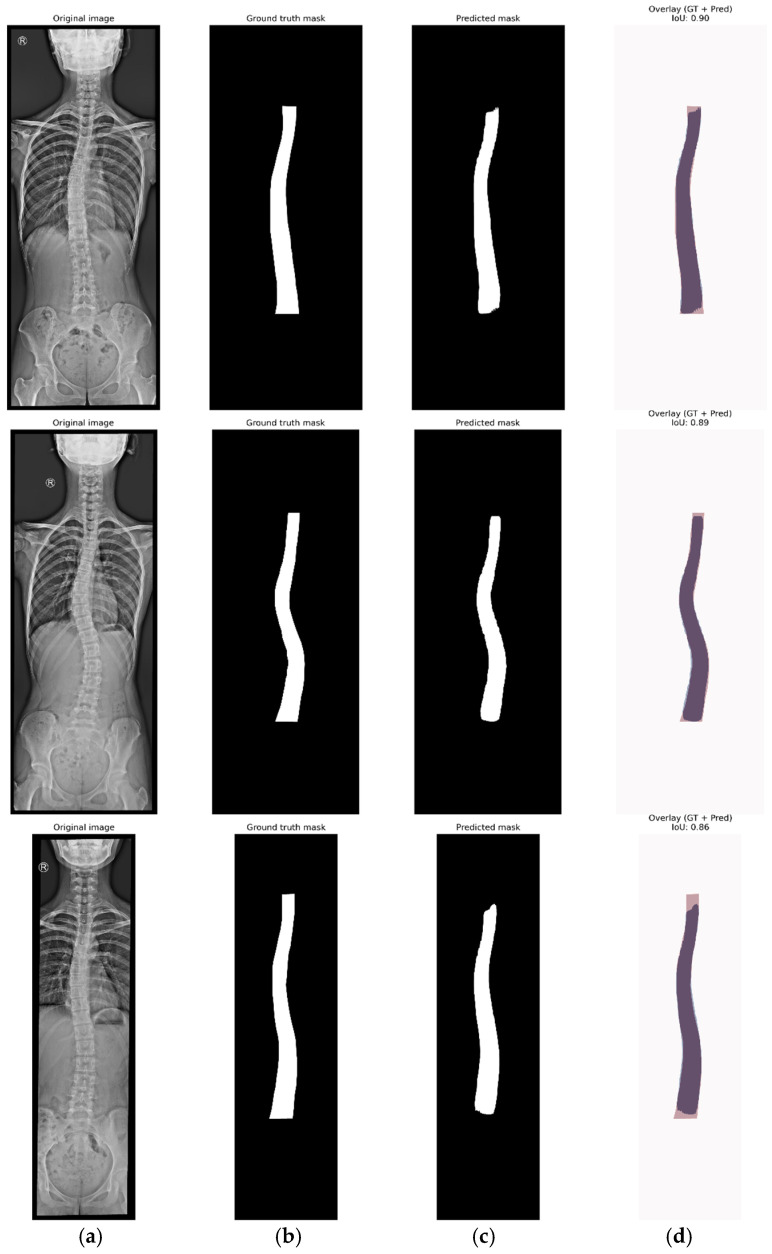
Images for (**a**) original image, (**b**) ground truth mask, (**c**) predicted mask, and (**d**) IoU.

The best epoch was determined on the validation loss function, reaching a minimum value of 0.3472 at epoch 287. Additionally, a comparative analysis was performed using different detection confidence thresholds, ranging from 0.70 to 0.90 in increments of 0.05, to identify the optimal segmentation accuracy in terms of mean IoU (mIoU) and mean AP (mAP). As a result, the highest mIoU metric was achieved at epoch 146, indicating the most accurate segmentation adjustment, while epoch 155 provided a slightly higher value in overall detection accuracy (mAP). Given that our approach focuses on extracting the spine contour from the mask generated by the Mask R-CNN network, we consider the mIoU metric as a key indicator of the agreement between the manually annotated mask (ground truth) and the predicted one. The analysis also revealed that a minimum detection confidence value of 0.85 provided the best segmentation result. To demonstrate network’s reliability and robustness, even with a limited dataset, we assessed the discrepancies between the predicted masks and the ground truth, which consisted of the annotated images in VGG Annotator. We employed intersection over union (IoU) to evaluate segmentation accuracy. Figure 5 illustrates the method used to calculate the mean IoU by processing the original image, the ground truth mask previously identified and annotated, the predicted mask, and the corresponding IoU values of several X-ray images.

#### 3.2.3. Processing Stage 2: Cobb Angle Quantification and Scoliosis Severity Assessment

**Figure 6:**
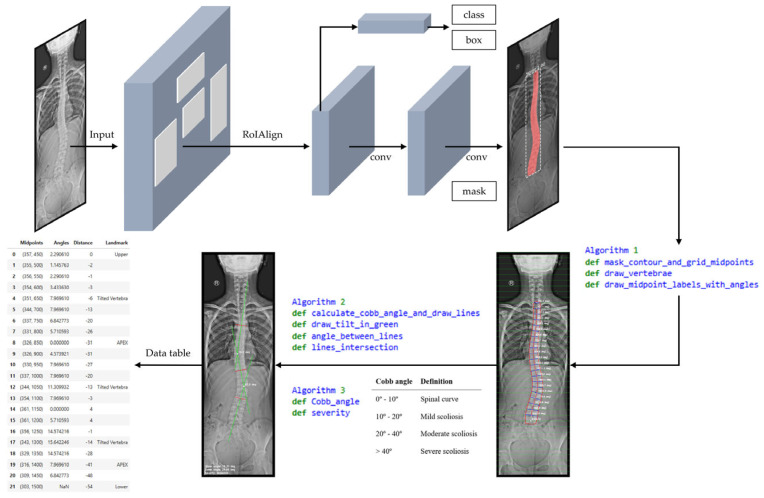
Detailed inference workflow for scoliosis assessment.

Once the mask was generated, we applied various algorithms developed to identify key anatomical landmarks along the spine boundary. The model calculated the position of the most tilted vertebrae, which allowed the quantification of the Cobb angle and the classification of scoliosis severity directly on the original image. Figure 6 shows a depiction of this stage, supported by the representation using the weights from the best epoch identified in the previous stage. The input AP X-ray image, corresponding to the test subset, was automatically rescaled to a height of 2000 pixels.

**Figure 7:**
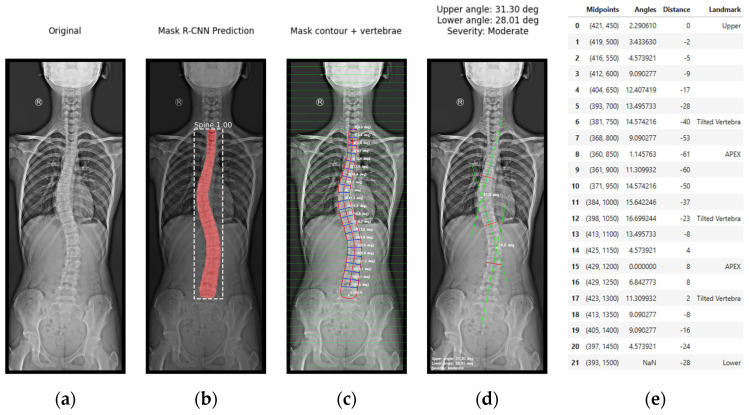
Visualization of the stages in JupyterLab: (**a**) original anteroposterior (AP) full spine X-ray image; (**b**) Mask R-CNN spine segmentation; (**c**) mask contour and vertebral angles; (**d**) computed Cobb angle, and severity classification (red lines correspond to the most tilted vertebrae, while green lines represent the perpendiculars used in the Cobb angle calculation); (**e**) data table displaying numerical values and anatomical landmarks.

The postprocessing stage requires the visualization of the results. To this end, we developed an interface in JupyterLab. Figure 7 presents a visualization of the results obtained during the inference workflow within the JupyterLab environment. This workflow operates as follows:

- Image acquisition is visualized in Figure 7a. The process begins with image acquisition through a user interface that allows the upload of an anteroposterior (AP) full spine X-ray image.
- Figure 7b shows the spine segmentation step using Mask R-CNN. Before inference, the input image is rescaled to a height of 2000 pixels. Once the model is loaded, the inference is initiated on the input image. We defined the confidence value during training. Only masks with a confidence score above this threshold are considered as detected regions. The mask with the largest segmented area is selected as the spinal region, assuming it corresponds to the spine. The Cobb angle quantification is highly dependent on the precision of the generated mask. The more accurate the mask, the more precise the assessment. To this end, the prediction of the model was optimized during training, and the best epoch was used for inference, aiming to ensure the highest possible accuracy in the generation of the mask.
- In Figure 7c, contour extraction and midpoint detection are illustrated. Once the segmentation mask is obtained, the spinal contour is extracted using OpenCV’s cv2.findContours() function, which is employed for contour detection in binary images. This contour with the largest segmented area is overlaid onto the image. Our approach is based on the capacity of the algorithms developed to build the spinal curvature within the contour extracted. To this end, we proposed the use of a grid, only considering the horizontal lines drawn in the image, to establish points that will be used to connect and build the spinal curvature. The interface provides a widget to define the grid interval, offering flexibility during the adjustment process. The objective of this method is to ensure that these horizontal lines fall within the lumbar and thoracic vertebrae with the highest possible accuracy. This technique facilitates an approximation of individual vertebrae segmentation. Each vertebra is segmented, without the need to train the model to detect each vertebra individually. The optimal grid interval value, predefined to 50 pixels, was defined through experimental testing using the widget and observing that the estimation of the midline spine curvature replicates the contour shape. At each grid line, two intersection points are detected where the line crosses the spinal contour. The algorithm calculates the mean distance between these two points, defining the midpoint on the image. These midpoints are the key reference for spinal curve construction through their connection. The extracted midpoints approximate the spinal curve, with the first and last point identified as upper and lower, limiting the length of the spine. A spline interpolation is applied to refine the connections between midpoints, smoothing the spinal path. Then, the algorithm draws a line perpendicular to the tangent of the curve at each midpoint, excluding the upper and lower points. The angle between these perpendicular lines with respect to the horizontal represents the vertebrae inclination at each midpoint and is computed and annotated on the image. The algorithm is designed to draw these tilted lines within the contour as a simplified representation of the vertebrae. This approach just described, which enables the spinal curvature estimation, has been designed as a proof of concept for the proposed methodology. It clearly depends on prior segmentation and assumptions such as local vertebral symmetry. However, these elements are part of an improvement process, and its optimization further strengthens the results of this initial proposal.
- Figure 7d depicts spinal curve estimation and Cobb angle calculation, based on the analysis of the curvature. The algorithm swipes the curvature and identifies the key anatomical landmarks following their definition: tilted vertebrae are defined as the vertebrae with the greatest inclination angle. They represent the greatest spinal deviation. Apex points correspond to the locations where the spinal curvature reaches its maximum deformation; that is, the points with the greatest lateral displacement relative to a vertical reference line. In our study, this reference is defined as the vertical line drawn from the upper reference point. It is important to note that when the algorithm addresses complex curvatures, two apex points are detected, one on the left (corresponding to negative distances), and one on the right (corresponding to positive distances). The algorithm was designed with a logic to distinguish the type of the curvature, simple or complex, in order to detect the correct number of tilted vertebrae and apex points, to provide either a single Cobb angle measurement or separate upper and lower Cobb angle measurement. The apex point, which refers to the peak of the spinal deviation, is critical for scoliosis assessment. To emulate the manual method, the most tilted vertebrae are isolated and represented on the image, remarked with red lines. Then, the algorithm draws perpendicular green lines from the midpoints corresponding to the most tilted vertebrae and connects them, as performed by clinical experts. The algorithm identifies the intersection point between these green lines, and the angle formed at their intersection is calculated. The Cobb angle and the severity classification are annotated on the image.
- Finally, the visualization and data export stage is presented in Figure 7e. The output is displayed in a multi-panel layout including the input X-ray image; the result of the Mask R-CNN segmentation; the extracted spinal contour with the computed midpoints, midline, and vertebral inclinations; the image showing Cobb angle measurement and severity classification; and the data table containing geometrical information, anatomical landmarks such as tilted vertebrae and upper, lower, and apex points. Processed images and the data table are exported as structured reports in .png and .csv formats, providing a comprehensive representation of the entire process.

The workflow detailed above provides clinicians with visual information to understand the process. By overlaying the Cobb angle procedure on the original image, it facilitates comparison with manual measurements, supporting decision-making in scoliosis assessment when necessary. Furthermore, the visualization of anatomical landmarks and results enhances transparency and can help experts in further evaluations.

#### 3.2.4. Postprocessing Stage: Agreement Between Manual and the Automated Cobb Angle Method

In this subsection, the agreement between manual and automated Cobb angle measurements is evaluated. The objective is to compare the measurements obtained by two observers and the model, by registering and analyzing the data collected from both sources.

**Figure 8:**
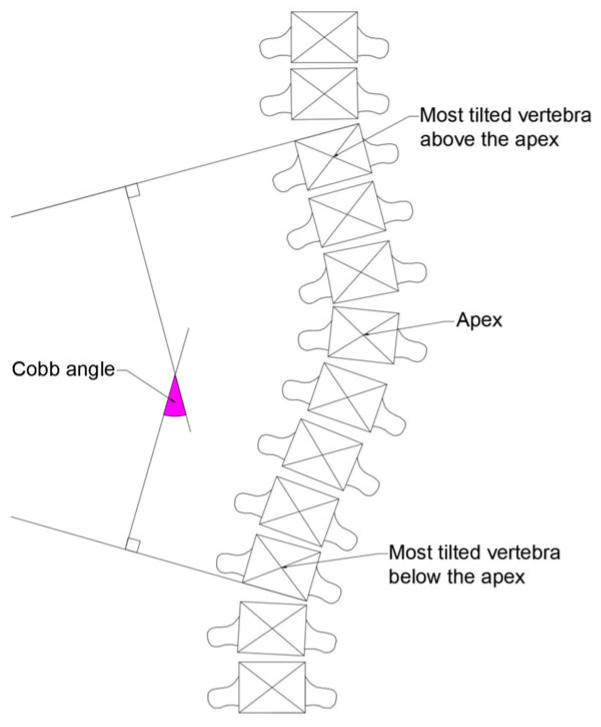
Scheme of Cobb angle measurement.

The manual procedure for Cobb angle measurement is described as follows. First, the observer identifies the apex of the spinal curvature. The apex is the vertebra that is most laterally displaced from the midline. Once the apex has been determined, the observer selects the most tilted vertebra above and below the apex. The observer draws two lines, one along the upper endplate of the most tilted vertebra above the apex, and the other along the lower endplate of the most tilted vertebra below the apex. As illustrated in Figure 8, the angle formed between these two lines, or between their perpendiculars if the lines do not intersect, is considered the Cobb angle [45]. Our approach has also been designed to evaluate the severity of scoliosis according to the classification shown in Table 1.

Once the Cobb angle manual procedure was finalized, a comparison with the automated method was designed. To this end, two experts, referred to as Observer A and Observer B, were asked to analyze the radiographs of the test set and calculate the Cobb angle using the aforementioned procedure. Having two observers allows for comparison of their measurements and reduce subjectivity, lowering uncertainty in the results. The measurements of both observers were then compared using various error metrics and Bland–Altman analysis, which is briefly described below:

- A Bland–Altman analysis [46] was carried out to evaluate the agreement between two methods by plotting the difference between the two measurements against their average. This graphical method allows the identification of any systematic bias and the definition of the limits of agreement. It is commonly used in medical applications to evaluate whether an automated method can replace or complement manual approaches.
- The intraclass correlation coefficient (ICC) with 95% confidence interval [47] is used to evaluate the reliability of measurements between two or more observers. In this case, it assessed the level of agreement between automatic and manual Cobb angle quantifications. The 95% confidence interval provides an estimate of the precision and stability of the ICC value.
- The median absolute difference (MAD) [47] is a robust measure of dispersion that is calculated as the median of the absolute differences from the median of any dataset. MAD is less affected by outliers than the standard deviation, making it a suitable tool for assessing variability when there are a few extreme values. In this case, it provided a typical error estimation that was more robust against outliers or occasional discrepancies.
- The mean absolute error (MAE) [47] was also included to quantify the average of the differences between the Cobb angles obtained manually and those obtained automatically.
- The standard deviation (SD) [47] measures the variability of the differences between measurements.

Both the MAE and MAD are presented with their corresponding standard deviation (±SD) to provide both a central estimate of the error and its variability. This combined presentation allows the assessment of how close the errors are to the mean and the detection of relevant dispersions.

## 4. Experimental Analysis and Results

In this section, the third research question of this study, “Is the proposed method accurate?”, is addressed. To answer it, the results obtained were evaluated through a comprehensive experimental analysis. First, the main results related to instance segmentation are presented. Second, a comparison between manual and automated Cobb angle measurements on AP X-ray images is provided using statistical analysis.

### 4.1. Instance Segmentation

A summary of the main metrics analyzed during the training phase is presented in Table 2, including the mean IoU, mean DSC, and mean AP (IoU = 0.5:0.95). In image segmentation, both IoU and DSC are commonly used to quantify the overlap between predicted and ground-truth regions, while mAP, calculated at multiple IoU thresholds, assesses the model’s precision and robustness across different overlap criteria. We also report over-segmentation and under-segmentation rates, which provide insights into how accurately the model delineates the target region. Epoch 146 achieved a 0.8012 mIoU, 0.8878 mDSC, and 0.6450 mAP, with over-segmentation and under-segmentation values of 0.0855 and 0.1357, respectively, indicating a favorable balance between precision and recall in the model’s spine segmentation performance.

Notably, the highest mIoU metric was achieved at epoch 146 with a minimum detection confidence of 0.85. This mean IoU score indicates strong segmentation performance, although there remains ample room for further improvement.

### 4.2. Cobb Angle Measurement

Accuracy in Cobb angle measurements and scoliosis severity was evaluated through a comparison between manual measurements and the output of the automated method. Manual measurements were performed independently by two separate observers, both familiar with the Cobb angle method. The procedure consisted of identifying on the X-ray images the apex and the most tilted vertebral endplates and then measuring the Cobb angles between those vertebrae. Conventional instruments, such as a ruler and a goniometer, were used during this process. All manual measurements were annotated on the radiographs and correctly classified into their corresponding subsets: 70% for training, 15% for validation, and 15% for testing. Only the measurements included in the testing subset were compared with the automated results.

As mentioned in Section 3, the algorithm developed in this study, inspired by the manual Cobb angle measurement procedure, provides anatomical landmarks, Cobb angle measurements, and severity classification. This process is based on the performance of Mask R-CNN for instance segmentation and its capability to generate a mask with the shape of the region of interest, in our case, the spine. In the following step, the contour of the mask is extracted, and the algorithm applies a set of rules to perform all measurement tasks, including anatomical landmark identification.

While the observers identified the apex and the most tilted vertebrae, as did the automated method, only the Cobb angle values were compared in this study. This opens the opportunity for future comparisons regarding anatomical landmark localization, aiming to improve the assessment of interobserver and automated agreement.

The degree of consistency between observers was assessed using the ICC with a 95% CI, and variability was evaluated using MAD ± SD and MAE ± SD (see Table 3 for definitions).

The results are presented in Table 3, showing an interobserver agreement of 0.939 (95% CI: 0.868–0.971), which is considered an excellent level according to the classification provided by [41]. The MAD and MAE values were 3.00°± 1.67° and 3.31° ± 1.53°, respectively, both providing the magnitude and dispersion of the absolute differences between observers.

**Figure 9:**
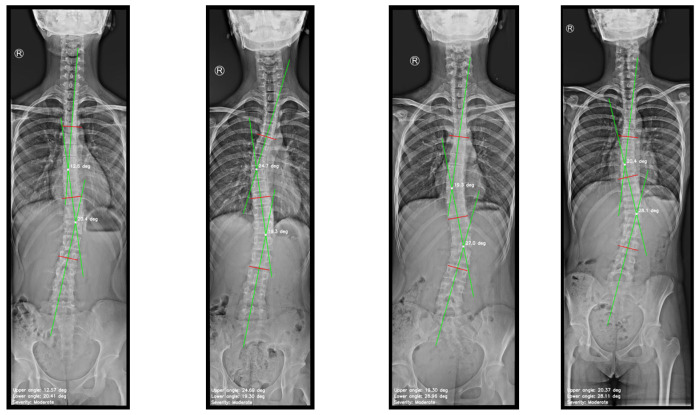

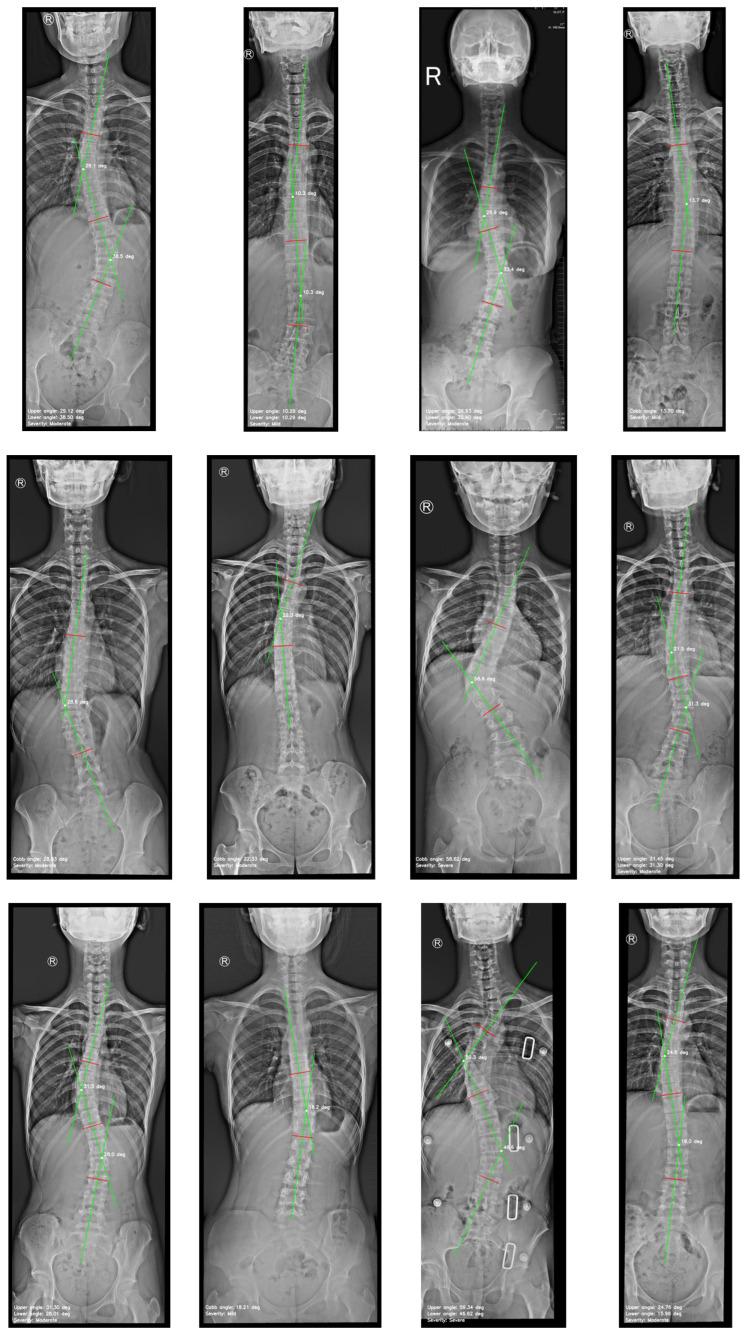
Images generated by the automated approach displaying the scoliosis assessment.

The variability between the results predicted by the model and the ground truth, specifically the manually measured Cobb angles, was compared across multiple X-ray images. Figure 9 shows the model predictions on the X-ray images, which were used to assess this discrepancy.

We employed statistical methods to evaluate the agreement between manual and automated Cobb angle measurements. They included Bland–Altman analysis, ICC with 95% CI, MAD, MAE, and SD.

A summary of the results is presented in the following tables. Table 4 reports the average Cobb angle measurements for both observers and the automated approach, while Table 5 summarizes the agreement between manual and automated methods and the errors.

**Figure 10:**
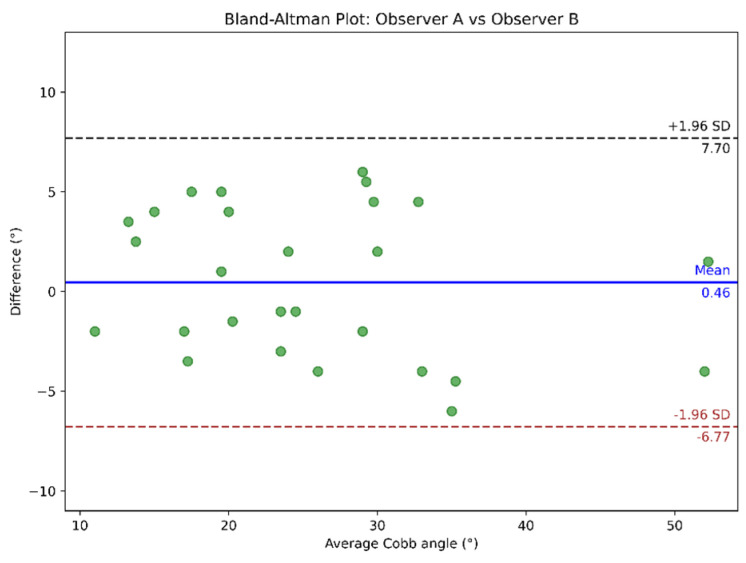
Bland–Altman plot comparing Cobb angle measurements from Observers A and B.

**Figure 11:**
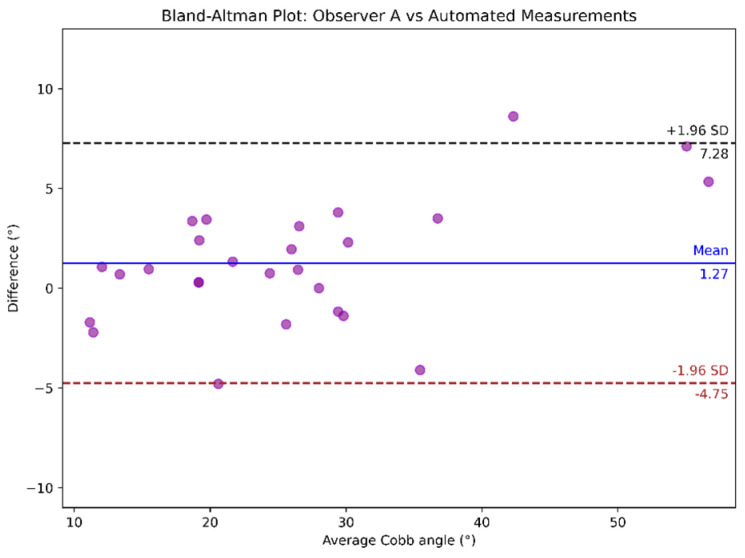
Bland–Altman plot comparing the automated vs. Observer A’s Cobb angle measurements.

**Figure 12:**
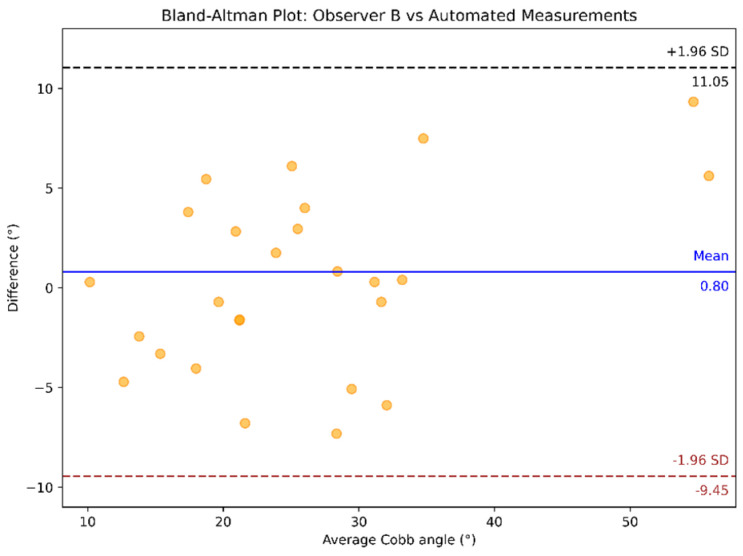
Bland–Altman plot comparing the automated vs. Observer B’s Cobb angle measurements.

Bland–Altman analysis was conducted to compare the measurements between observers, as shown in Figure 10, and between observers and automated Cobb angle method, as illustrated in Figure 11 and Figure 12. In the Bland-Altman plots, the x-axis represents the average Cobb angle between the two methods being compared. The y-axis shows the difference between their measurements. Each dot corresponds to an individual measurement pair. The blue line indicates the mean of the difference (bias), and the dashed black and brown lines represent the limits of agreement.

Notably, in Figure 11, the outlier corresponds to a difference of 8.62 degrees between the manual measurement (Observer A) and the model’s prediction. Upon inspection of the corresponding image, we observed that the model produced a simplified representation of one vertebra, with greater inclination than the actual. This example of overestimation of the measured angle explains the discrepancy.

## 5. Discussion and Scope

In this research, the authors propose an automated approach for scoliosis assessment, which is divided into two processing stages. First, the Mask R-CNN architecture was trained for the spine segmentation within a dataset that comprised anteroposterior X-ray images of the spine. The convolutional neural network generates a mask that provides the starting point for the second component, in which a set of algorithms extracts the mask contour of the spine by identifying anatomical landmarks, enabling Cobb angle quantification and scoliosis severity assessment. During inference, the input images, unseen by the network, are rescaled to a height of 2000 pixels, the same height as those in the dataset. This allows the integration of a rescaling layer for any new image to be evaluated.

It is important to note that the neural network processes the annotations provided in the dataset, which correspond to the spinal region. During training, the network learns to identify this region based on the annotations. These labels are manually annotated only once per image. However, if new images are added to the training dataset, they must be rescaled, annotated, and distributed to the subset before starting the training process that includes the new images. The dataset used in this study was classified as imbalanced, with more than 50% of the cases considered moderate.

Although this class imbalanced is not a classification objective of the network, it may affect the model’s generalization capability. In general terms of robustness, a segmentation network such as Mask R-CNN may perform better on imbalanced datasets than a classification model, particularly in this case, where the task concerns scoliosis severity.

This approach focuses on the accurate detection and segmentation of the spinal region within X-ray images. If the task is the identification of scoliotic curvatures, it is reasonable to assume, even with the current imbalance, that the network’s generalization is not considerably affected. The application of transfer learning described in Section 3.2.2 supports this comment. We employed a pre-trained network in a similar application, the detection of objects, such as the spine in this case, to mitigate the imbalance in the dataset. This particular feature enables the network to use its previous knowledge to focus on segmentation. As a result, the model becomes less dependent on a well-balanced dataset and more robust against potential bias caused by imbalance.

Nevertheless, a high imbalance could introduce biases that may affect the network’s detection capability or even distort the shape of the segmented region. Therefore, the dataset imbalance should be reviewed, addressed, and corrected, even if the network is less sensitive to these effects and its greater reliability in the identification of anatomical regions.

We only considered the spine class label, while the output is limited to the spine localization and the mask generation. A set of algorithms during the inference stage quantifies the Cobb angle and assesses the scoliosis severity. The objective of analyzing the degree of spinal curvature is to evaluate the error between manual and automated measurements, particularly using the testing subset, which may support future research.

Our method uses the mask generation capability of Mask R-CNN and takes advantage of this feature by applying a single-instance segmentation approach instead of vertebrae segmentation. We reduced the annotation procedure and computational cost by considering the spine structure as a single object, resulting in larger masks that may facilitate the learning process. We also performed monitoring of the segmentation metrics, which is a highly relevant practice for identifying the most suitable candidate epoch. The authors recommend this evaluation to enhance the understanding of the training process and to achieve the highest possible network performance.

Network instance segmentation and mask generation evaluation achieved an mIOU of 0.8012, mDSC of 0.8878, and mAP of 0.6450. Although a larger dataset is required to improve segmentation, the results obtained confirm that Mask R-CNN presents a reliable performance in spine detection and segmentation.

OpenCV’s cv2.findContours() function plays a fundamental role in detecting and extracting the spinal contour from the generated mask. This contour enables users to draw the midline of the spine, which is relevant for Cobb angle calculation. The midpoints technique provided in this study define key references along the spinal curvature, allowing users to emulate vertebrae without the need to represent each vertebra individually. The function of the grid interval, which is designed to place a series of points within each vertebra, provides its inclination. The rescaling layer, which adjusts input images to a height of 2000 pixels, ensures the functionality of the grid interval.

We achieved good agreement in Cobb angle measurements with a grid interval value of fifty pixels, which was defined through experimental testing. However, users may adjust the grid interval using the implemented widget. This set of techniques enables the depiction of the spinal midline, aiming to replicate its curvature. A spline interpolation smooths the trajectory defined by the midpoints, providing a continuous representation of the spine. The curvature is then analyzed as a continuous mathematical function by examining its behavior. This analysis enables the estimation of the anatomical landmarks required for Cobb angle quantification.

The accuracy of Cobb angle quantification was assessed through several statistical metrics. Our automated approach achieved an MAD ± SD of 2.17° ± 2.51°, an MAE ± SD of 2.96° ± 2.60°, and an ICC (95% CI) of 0.928 between observers and the automated method. The inference workflow required an average time of 3.3 s per image, providing four images with the step of the process overlaid onto them. This result demonstrates the low deviation of our approach in the Cobb angle quantification task, which is comparable to the measurements performed by observers.

The proposed method, as presented and discussed in this study, can, and must be classified as automatic, despite the initial labelling stage for the dataset. As mentioned in the Material and Methods section, this preliminary manual annotation must be carried out on the X-ray images so that the convolutional neural network can correctly recognize the regions corresponding to the spine. This involves placing points along the contour of the spine. Based on these annotations, the original radiograph is converted into a binary representation in which the spine appears in white and the background in black. This converted image is used by the network as the training label, with the corresponding rescaled X-ray images serving as inputs. While this is a manual and preliminary step, once completed, and with the network properly trained and optimized, the process of Cobb angle quantification for new anteroposterior radiographs becomes fully automated, with no need to manually place points again. For this reason, it is reasonable to describe the method as automatic.

Although the results obtained were promising, the authors acknowledge that various aspects require a critical examination. On the one hand, as previously mentioned, the limited size of the dataset may affect the model’s generalization when increasing the number of patients. On the other hand, our single-instance segmentation approach requires a precise contour representation. Small errors in contour detection or non-smooth boundaries may misalign midpoints, thereby affecting the accuracy of Cobb angle quantification. While the fixed grid interval, midpoint technique, and spline interpolation are efficient, the method assumes a typical scoliotic anatomy. In cases of severe or atypical deformities, limited in number within the dataset, the Region Proposal Network (RPN) may generate a bounding box that is too narrow to capture the full lateral extent of the spine. This may crop the mask, excluding peripherical regions, thereby producing incomplete representation that could result in an inaccurate curvature reconstruction affecting the midline extraction. In addition, the requirement for high-quality X-ray images restricts the applicability of this approach in more realistic clinical environments, where images with noise or complex projections, such as overlapping anatomical structures or unusual patient rotations, may be encountered.

Finally, in a real scenario, providing a complete overview of the workflow may enhance the expert’s understanding of the method, support clinical diagnosis, and enable the storage of results for monitoring scoliosis progression in patients.

## 6. Conclusions

This study enabled the authors to address the three main research questions: “Where is the spine in this radiograph?”, “What is its exact shape?”, and “Is the proposed method accurate for Cobb angle measurement?”. Mask R-CNN answered the first two questions: the spine localization within the image (where), and the shape definition of the spine (what). The third question was answered by a set of algorithms specifically designed to achieve accuracy in Cobb angle quantification and severity classification.

The Mask R-CNN network under a single-instance segmentation approach and the midpoint-based method applied in this study enabled the extraction of the scoliotic curve’s midline and the identification of tilted vertebrae necessary for Cobb angle estimation, achieving a strong agreement between automated and manual measurements. Notably, the choice of segmenting the spine as a single object involves single-class annotation (spine plus background), which helps mitigate the impact of dataset imbalance, as the network’s task is not based on the Cobb angle classification.

The authors acknowledge that the limited size of the training dataset constitutes the main limitation of this study, compromising the model’s ability to generalize to unseen cases. Factors such as increased patient variability, overlaid structures, or low-resolution images during inference, may affect the performance of the proposed model. This could impact the Cobb angle estimation, especially in cases where a measurement error of a few degrees could alter the severity assessment. In this regard, our approach provides both Cobb angle quantification and severity assessment, emphasizing the angle value rather than the severity to facilitate data-driven clinical decision-making.

The agreement between manual and automated measurements obtained in this study demonstrates that our approach achieves the objective of offering an automated method for Cobb angle quantification and scoliosis severity classification, with results comparable to those estimated by human observers. The indicators used for accuracy assessment suggest that the model may be acceptable for many clinical scenarios. Nevertheless, given that this study was conducted using a limited sample of images, more extensive external validation is required to verify its robustness. Our method provides experts with a clear visualization of each stage in the scoliosis assessment. This enhances the interpretability of the results, facilitates monitoring, and enables comparison when experts evaluate the scoliosis progression.

This study is within the scope of a broader research line. As a part of our future work, we plan to explore strategies such as data augmentation and vertebrae segmentation, and to compare the results of this study with those of other approaches, aiming to improve model generalization.

## Figures and Tables

**Table 1:** Cobb angle definition [2].

Cobb Angle	Definition
0–10°	Spinal curve
10–20°	Mild scoliosis
20–40°	Moderate scoliosis
>40°	Severe scoliosis

**Table 2:** Summary: mean IoU (mIoU); mean dice similarity coefficient (mDSC); mean average precision (mAP, IoU = 0.5:0.95); mean precision; mean recall; over-segmentation; under-segmentation.

Threshold	mIoU	mDSC	mAP	Mean Precision	Mean Recall	Over-Seg	Under-Seg
0.85 (epoch 146)	0.8012	0.8878	0.645	0.9145	0.8643	0.0855	0.1357
0.85 (epoch 155)	0.7980	0.8857	0.655	0.9150	0.8599	0.0850	0.1401
0.85 (epoch 287)	0.7818	0.8750	0.625	0.9313	0.8268	0.0687	0.1732

**Table 3:** Interobserver agreement in manual Cobb angle measurement method.

Analysis	ICC (95% CI)	MAD ± SD	MAE ± SD
Observer A vs. Observer B	0.939 (0.868, 0.971)	3.00° ± 1.67°	3.31° ± 1.53°

ICC: intraclass correlation coefficient; CI: confidence interval; MAD: median absolute difference; MAE: mean absolute error (MAE); SD: standard deviation.

**Table 4:** Summary of manual and automated Cobb angle measurements on AP X-ray images.

Cobb Angle Measurements	Mean ± Standard Deviation (Range)
Manual measurement by observer A	25.43° ± 10.85° (range 11.50–54.00°)
Manual measurement by observer B	25.89° ± 10.00° (range 10.00–53.00°)
Measured by the automated method	26.69° ± 12.50° (range 10.29–59.34°)

**Table 5:** Summary of the agreement between manual and automated methods.

Analysis	ICC (95% CI)	MAD ± SD	MAE ± SD
Observer A vs. observer B	0.939 (0.868, 0.971)	3.00° ± 1.67°	3.31° ± 1.53°
Observer A vs. automated	0.961 (0.926, 0.984)	2.15° ± 2.03°	2.54° ± 2.06°
Observer B vs. automated	0.895 (0.780, 0.950)	3.60° ± 3.27°	4.07° ± 3.22°
Overall: Observer A & B vs. automated	0.928 (0.853, 0.967)	2.17° ± 2.51°	2.96° ± 2.60°

## Data Availability

Data are contained within the article.
